# Geriatric influenza death (GID) score: a new tool for predicting mortality in older people with influenza in the emergency department

**DOI:** 10.1038/s41598-018-27694-6

**Published:** 2018-06-18

**Authors:** Jui-Yuan Chung, Chien-Chin Hsu, Jiann-Hwa Chen, Wei-Lung Chen, Hung-Jung Lin, How-Ran Guo, Chien-Cheng Huang

**Affiliations:** 1 Department of Emergency Medicine, Cathay General Hospital, Taipei, Taiwan; 2 Department of Emergency Medicine, Chi-Mei Medical Center, Tainan, Taiwan; 3 Department of Biotechnology, Southern Taiwan University of Science and Technology, Tainan, Taiwan; 4 Fu Jen Catholic University School of Medicine, Taipei, Taiwan; 5 Department of Emergency Medicine, Taipei Medical University, Taipei, Taiwan; 6 Department of Environmental and Occupational Health, College of Medicine, National Cheng Kung University, Tainan, Taiwan; 7 Department of Occupational and Environmental Medicine, National Cheng Kung University Hospital, Tainan, Taiwan; 8 Department of Senior Services, Southern Taiwan University of Science and Technology, Tainan, Taiwan; 9 Department of Geriatrics and Gerontology, Chi-Mei Medical Center, Tainan, Taiwan; 10 Department of Occupational Medicine, Chi-Mei Medical Center, Tainan, Taiwan

## Abstract

Although influenza may cause death in the geriatric population, the best method for predicting mortality in this population is still unclear. We retrospectively recruited older people (≥65 yr) with influenza visiting the emergency department (ED) of a medical center between January 1, 2010, and December 31, 2015. We performed univariate and multivariate logistic regression to identify independent mortality predictors and then developed a prediction score. Four hundred nine older ED patients with a nearly equal sex ratio were recruited. Five independent mortality predictors were identified: severe coma (Glasgow Coma Scale score ≤8), past histories of cancer and coronary artery disease, elevated C-reactive protein levels (>10 mg/dl), and bandemia (>10% band cells). We divided the patients into three mortality risk and disposition groups: (1) low risk (1.1%; 95% confidence interval [CI], 0.5–3.0%); (2) moderate risk (16.7%; 95% CI, 9.3–28.0%); and (3) high risk (40%; 95% CI, 19.8–64.2%). The area under the receiver operating characteristic curve and the Hosmer-Lemeshow goodness of fit of the GID score were 0.86 and 0.578, respectively. The GID score is an efficient and simple tool for predicting mortality in older ED patients with influenza. Further studies are warranted to validate its use.

## Introduction

The rapidly ageing population is a global issue. In the United States, the older population (aged ≥65 yr) is estimated to reach 89 million people by 2050, potentially constituting around 28% of the total U.S. population^1^. Taiwan has one of the most rapidly aging populations in the world^2,3^. The geriatric population in Taiwan in 2017 represents 13.3% of the country’s total population, and it is estimated that it will grow to 20% by 2025^2,4,5^. This aging population has a significant impact on the health care system. In 2015, 38.5% of Taiwan National Health Insurance expenditures were contributed by the geriatric population^6^. A recent study in Taiwan demonstrated that older people also account for 35.6% of emergency medical services calls^7^.

Influenza is a common cause of death in older people^8^. The morbidity and mortality caused by influenza are often attributed to secondary bacterial infections and complications^8^. Although influenza seasons may vary in severity, the older population is most vulnerable to severe influenza^9^. A 2004 study in the U.S. reported that the older population accounted for about 186,000 excess hospitalizations^10^. Another study in the U.S. in 2003 reported that older persons accounted for 44,000 excess deaths resulting from all causes^11^. During influenza seasons, deciding whether to admit older person with influenza to the hospital is very difficult because of limited medical resources; therefore, predicting mortality in older people with influenza and their subsequent disposition become very important issues. Although researchers in several studies have reported mortality predictions for, and the critical care resource use of patients with influenza, most of these studies were focused on mortality prediction for adults in general, the prediction of specific influenza subtypes, and prediction of hospitalization^12–14^. Mortality prediction for older people with all subtypes of influenza is still unclear. Therefore, we conducted this study with the aim of delineating this issue.

## Results

In total, we recruited 409 patients for this study. The mean age (±SD) was 79.5 (±8.3) yr, and the sex ratio was nearly equal (Table 1). The most common past histories were hypertension (64.3%), diabetes (39.8%), chronic obstructive pulmonary disease (27.1%), and coronary artery disease (25.1%). In the analysis of subtypes, influenza A was dominant (68%), whereas coinfection with influenza A and B comprised 2.7% of subtypes. The most common complications of influenza were pneumonia (67.5%), urinary tract infection (18.1%), and sepsis (8.3%). Admission and 30-day mortality rates were 83.9% and 4.9%, respectively. All patients were treated with either oseltamivir (94%) or zanamivir (6%) immediately when influenza was diagnosed in the emergency department (ED). Because the patients who had been treated in other hospitals were excluded, the recruited patients were treated with the antivirals for the first time during this influenza episode.

**Table 1:** Characteristics of geriatric emergency department patients with influenza.

Characteristics	Total patients (n = 409)
Age, yr	79.5 ± 8.3
Age subgroup	
Young elderly (65–74 yr)	30.6
Moderately elderly (75–84 yr)	42.5
Old elderly (≥85 yr)	26.9
Male sex	50.1
Vital signs	
GCS	13.9 ± 2.32
Severe coma (GCS ≤8)	5.1
SBP (mmHg)	146.1 ± 30.5
Hypotension (SBP <90 mmHg)	3.4
Heart rate (beats/min)	98.8 ± 20.5
Respiratory rate (breaths/min)	21.2 ± 4.1
Tachypnea (respiratory rate >20)	37.1
Tympanic temperature (°C)	38.1 ± 0.9
Past history	
Hypertension	64.3
Diabetes	39.8
Chronic obstructive pulmonary disease	27.1
Coronary artery disease	25.1
Bedridden	16.3
Stroke	15.8
Cancer	14.9
Congestive heart failure	9.0
Feeding with nasogastric tube	6.6
Nursing home resident	4.4
Dementia	2.2
Laboratory data	
WBC (cells/mm^3^)	10,590.0 ± 5820.0
Leukocytosis (WBC >12,000 cells/mm^3^)	43.5
Bandemia (band form >10%)	10.2
Hemoglobin (mg/dL)	12.1 ± 2.1
Anemia (hemoglobin <12 mg/dL)	17.1
Platelet (10^3^/mm^3^)	186.2 ± 158.8
Thrombocytopenia (platelet count <150 × 10^3^/mm^3^)	0.73
Serum creatinine (mg/dL)	1.6 ± 1.4
Renal impairment (serum creatinine >2 mg/dL)	17.8
CRP (mg/dL)	8.2 ± 10.1
Elevated CRP (>10 mg/dL)	32.2
Influenza subtypes	
Influenza A	68.0
Influenza B	29.3
Influenza A + B	2.7
Influenza vaccination	23.2
Complications*	
Pneumonia	67.5
Urinary tract infection	18.1
Sepsis	8.3
Admission rate^†^	83.9
30-day mortality rate	4.9

Data were presented as % or Mean ± SD. ED, Emergency Department; SD, standard deviation; GCS, Glasgow coma scale; SBP, systolic blood pressure; WBC, white blood cell count; CRP, C-reactive protein.

*Not all the complications are listed in the table.

^†^Admission to general ward or intensive care unit.

Univariate logistic regression analysis yielded ten univariate mortality predictors, each with a significance of *p* < 0.1: tachypnea, severe coma, hypertension, coronary artery disease, cancer, bedridden, leukocytosis, bandemia, anemia, and elevated C-reactive protein levels (CRP) (Table 2). Multivariate logistic regression analysis identified five independent mortality predictors: severe coma, elevated CRP, cancer, coronary artery disease, and bandemia. Specific points for each predictor were assigned to develop the geriatric influenza death (GID) score (Table 3).

**Table 2:** Univariate mortality predictors among geriatric emergency department patients with influenza.

Variable	Variable present		*p*-value
	Yes	No	
	n (% mortality)	n (% mortality)	
Tachypnea (respiratory rate >20)	152 (7.2)	257 (3.5)	0.09
Severe coma (GCS ≤8)	22 (22.7)	387 (3.8)	0.05
Hypertension	263 (6.4)	146 (2.0)	0.05
Coronary artery disease	94 (10.6)	315 (3.1)	<0.01
Cancer	61 (11.4)	348 (3.7)	0.01
Bedridden	67 (8.9)	342 (4.0)	0.09
Leukocytosis (WBC >12,000 cells/mm)	117 (7.6)	292 (3.7)	0.09
Bandemia (>10% band)	26 (11.5)	383 (4.4)	0.01
Anemia (hemoglobin <12 mg/dL)	163 (7.9)	246 (2.8)	0.02
Elevated CRP (>10 mg/dL)	98 (9.1)	311 (3.5)	0.02

ED, emergency department; GCS, Glasgow coma scale; WBC, white blood cell count; CRP, C-reactive protein. Variables with *p* < 0.1 in the analysis are shown.

**Table 3:** Independent mortality predictors by multivariate logistic regression analysis in the geriatric emergency department patients with influenza.

Variable	Parameters				GID score Points
	B	Bootstrapped OR	Bootstrapped 95% CI	*p*-value	
Constant	−5.25				
Severe coma (GCS ≤8)	3.04	4.57	2.29−9.11	<0.01	2
Elevated CRP (>10 mg/dl)	2.07	3.46	1.20−10.03	0.01	1
Cancer	1.94	6.96	2.19−22.06	0.02	1
Coronary artery disease	1.89	6.67	2.26−19.69	0.02	1
Bandemia (>10% band)	1.24	7.97	2.14−29.65	0.01	1
AUC				0.861	
Hosmer and Lemeshow Goodness of fit				0.578	
Possible scores in range				0−6	

ED, emergency department; OR, odds ratio; CI, confidence interval; GID, geriatric influenza death; GCS, Glasgow coma scale; CRP, C-reactive protein; AUC, area under the curve.

**Figure 1:**
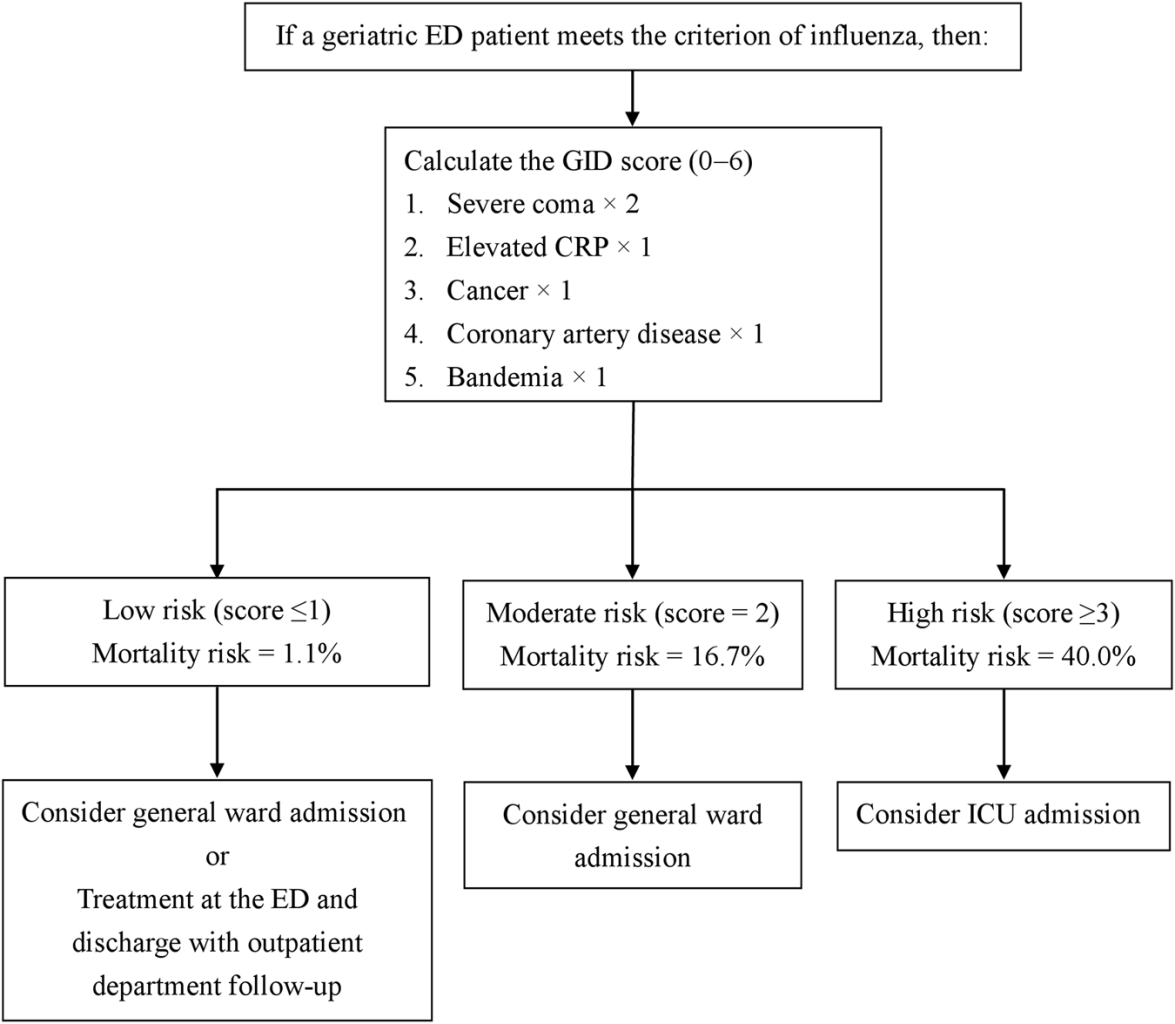
A suggested disposition based on the mortality risk determined by the GID score for geriatric ED patients with influenza. GID, geriatric influenza death; ED, emergency department.

**Figure 2:**
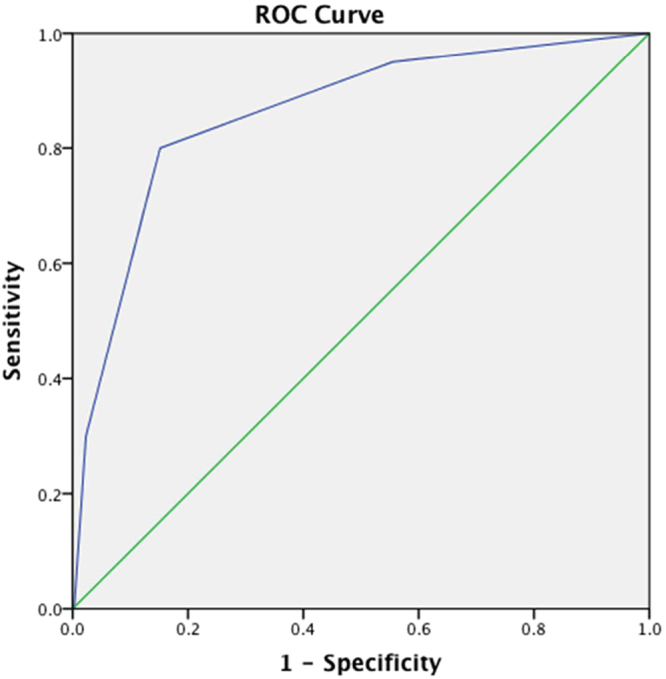
Area under the curve of the GID score. GID, geriatric influenza death.

According to GID score, the patients were divided into the following three risk subgroups: (1) low risk, defined as mortality risk of 1.1% (4/334; 95% confidence interval [CI], 0.5−3.0%); (2) moderate risk, defined as mortality risk of 16.7% (10/60; 95% CI, 9.3−28.0%); and (3) high risk, defined as mortality risk of 40.0% (6/15; 95% CI, 19.8−64.2%) (Fig. 1). The individual mortality risk for GID score is as follows: (1) score of 0, 0.6% (1/174); (2) score of 1, 1.9% (3/160); (3) score of 2, 16.7% (10/60); (4) score of 3, 42.9% (6/14); and (5) score of 4, 0% (0/1). Owing to the similar mortality risk of scores 0 and 1 and the small sample size of score 4, we combined scores 0 and 1 into the low-risk subgroup and scores 3 and 4 into the high-risk subgroup. The area under the receiver operating characteristic curve (AUC) of the GID score was 0.86 (95% CI, 0.77−0.94), which suggests excellent discrimination (Fig. 2). The Hosmer-Lemeshow goodness of fit was 0.578. The causes of death were pneumonia (15 [75%] of 20), bacteremia (2 [10%] of 20), and combined pneumonia and urinary tract infection (3 [15%] of 20). The classifications of influenza among the deaths were type A, 60% (12 of 20); type B, 25% (5 of 20); and types A and B, 15% (3 of 20).

## Discussion

We developed the GID score, consisting of five independent mortality predictors, to help predict 30-day mortality and suggested disposition of older people with influenza in the emergency department. The GID score had excellent discrimination and a good fit. After calculating the GID score, we found that high-risk patients with scores ≥3 are considered critically ill, and admission to the intensive care unit is suggested; moderate-risk patients with a score of 2 are recommended to be admitted to the general ward; and low-risk patients with scores ≤1 may be admitted to the general ward or discharged with outpatient department follow-up after ED treatment. This tool may help in determining how to effectively allocate medical resources and thus preserve them for patients with greater need. In addition, because the GID score was developed in the geriatric population with influenza by univariate and multivariate logistic regression analyses, it is more important and specific than the tools used for the overall geriatric population.

Severe coma is the strongest predictor of mortality in older ED patients with influenza. This finding is compatible with the Third International Consensus Definitions for Sepsis and Septic Shock, published in 2016, which suggest that altered mentation be one of the variables used to predict mortality among patients with infection (quick Sepsis-related Organ Failure Assessment [qSOFA] criteria)^15^. However, the state of altered mentation is often difficult to define, and should be compared with the patient’s baseline mental status. Using severe coma for the variable of mentation is more realistic and more feasible to quantify^16^. CRP is believed to be correlated with sepsis-related mortality. The researchers in a study that included 2785 adult patients in Denmark reported that CRP independently predicted 30-day mortality if the level was >10 mg/dl^17^. Researchers in another study also reported that risk of sepsis-related mortality appeared to have increased when the third-day CRP value was >10 mg/dl^18^. Researchers in a large nationwide study in the United States reported that patients with a history of cancer are at increased risk for acquiring sepsis and subsequently dying as a result^19^. Patients with cancer often are in an immunocompromised state due to the use of chemotherapy, radiation, or other immune-modulating therapy to combat the underlying malignancy^19^. They may also have impaired leukocyte function due to the malignancy itself, which makes them vulnerable to infection^19^. Influenza may lead to the exacerbation of preexisting cardiac disease and direct cardiac involvement, resulting in myocarditis and deterioration of heart failure^20^. The influenza virus is also assumed to play an important role in atherogenesis or atherothrombosis by destabilizing present vulnerable plaques, which may trigger acute myocardial infarctions and eventually result in an increased chance of mortality^21^. Bandemia is found to be a predictor of mortality in the diseases associated with infection^21,22^, and is one of the criteria for systemic inflammatory response syndrome (SIRS)^15^. Although the qSOFA has been emphasized as a better tool for identifying sepsis in recent studies, the SIRS criteria may remain useful for the identification of infection^15^.

We could not conclude that influenza vaccination was not effective despite this study showing a non-significant association between influenza vaccination and mortality. The sample is limited to older person presenting to the ED with influenza, thus providing a limited and potentially biased population with which to measure any association between influenza vaccine and outcome. In addition, the effectiveness of the influenza vaccine may varies from season to season^11,23^. The influenza vaccination plays an important role in attempts to reduce the mortality burden of influenza^11^. A previous study showed the benefits of inactivated influenza vaccine in preventing deaths resulting from complications of influenza infection^11^.

The prevalence of chronic obstructive pulmonary disease in this study was higher than in the overall geriatric population. A nationwide study in Taiwan reported that the prevalence of chronic obstructive pulmonary disease was around 8.8% in the population of ≥70 years in 2002^24^. The probable reason is that older persons with chronic obstructive pulmonary disease are more susceptible to influenza infections due to impaired pulmonary function^24^.

Despite this study’s strength of proposing a novel tool for predicting mortality in older ED patients, it has some limitations. First, some of the data may not have been collected completely, owing to the nature of a retrospective study design. Second, the results of this study may not be generalizable to other nations or other regions in Taiwan, owing to its being a single-center study. Further studies are warranted to validate the use of the GID score. Third, the influenza pharyngeal or nasal swabs used in this study—although the most practical choice for most medical facilities—are not a gold standard method with confirmed false-positive and false-negative rates. Further advanced examinations, such as reverse transcriptase–polymerase chain reaction, immunofluorescence assay, or viral culture, are needed to confirm the diagnosis of influenza. Fourth, some other laboratory markers, such as procalcitonin, lactic acid, and albumin, which are associated with mortality in patients with severe sepsis, were not included in this study. The reason why we did not include these measurements in our study is that it is not practical to perform these tests or to obtain these data for every older ED patient with suspected influenza. Fifth, there may be substantial differences in patients presenting within the influenza season and at during the off-season, because influenza is a seasonal illness. The variation between seasons is important, as different strains can have different severities for specific populations, such as older people. Therefore, subsequent studies about this issue are also warranted. Sixth, the susceptibility to antivirals was not included, which may affect the prognosis of influenza.

In conclusion, the GID score is an easy, simple, and efficient tool for predicting 30-day mortality and facilitating decision making for the disposition of older ED patients with influenza. The GID score also may potentially be applicable in other medical settings. Furthermore, using the ratio of the actual to expected number of deaths based on the GID score may help in the evaluation of the quality of care in older persons with influenza. Despite the advantages of the GID score, it cannot completely replace the clinical judgment of the treating physician.

## Methods

### Study design, setting, and participants

**Figure 3:**
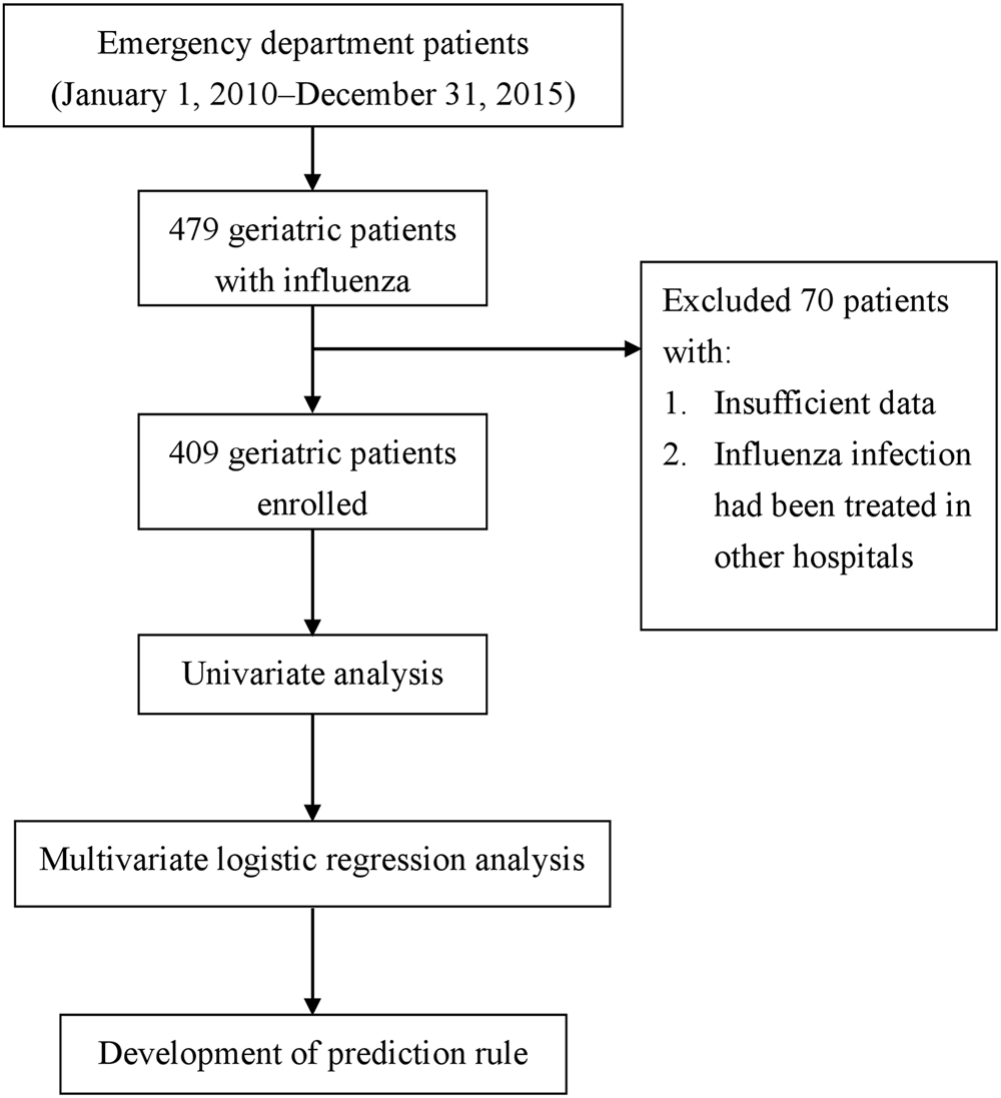
Flowchart of this study.

We conducted this study in an 825-bed, university-affiliated medical center in northern Taiwan. The center has a 40-bed ED providing care for approximately 55,000 patients per year^16,25^. Older people comprised about 33% of all ED patients. All older persons who visited the ED between January 1, 2010, and December 31, 2015, were recruited if they met both of the following criteria: (1) having a fever, defined as tympanic temperature ≥37.2 °C or a baseline temperature elevated ≥1.3 °C^16,25^; and (2) influenza infection, defined as a positive influenza pharyngeal or nasal swab result (either type A or type B)^26^. Overall, 479 older ED patients met the criteria for influenza. 409 patients were recruited after we excluded 70 patients who had insufficient data or had been treated in other hospitals. The recruited patients were divided into two groups on the basis of their 30-day outcome: survival vs. mortality. The study flowchart is shown in Fig. 3.

### Data sources, measurement, and definitions of the categorical variables

We retrospectively collected data, including demographic characteristics, vital signs, past history, laboratory data, complications, and outcomes, from medical records by an emergency physician. Information on the variables of interest for each patient was recorded (Tables 1 and 2). Any variable not recorded in the patient’s medical record was considered negative. We defined categorical variables as the following, in accordance with previous studies in emergency or geriatric medicine:

1. *Severe coma*: Glasgow Coma Scale score ≤8^16,27–29^.
2. *Bedridden*: Eastern Cooperative Oncology Group performance status 4 (completely disabled, cannot carry on any self-care, and totally confined to bed or chair)^25,30^.
3. *Hypotension*: Systolic blood pressure <90 mmHg^25,31^.
4. *Tachypnea*: Respiratory rate >20/min^25^.
5. *Leukocytosis*: White blood cell count >12,000 cells/mm^3^ ^16,25,31^.
6. *Bandemia*: Immature band form count >10%^15^.
7. *Anemia*: Hemoglobin <12 mg/dl^32^.
8. *Thrombocytopenia*: Platelet count <150 × 10^3^/mm^3^ ^33^.
9. *Renal impairment*: Serum creatinine >2 mg/dL^27^.
10. *Elevated CRP*: >10 mg/dL^34^.

Complications of influenza include pneumonia, urinary tract infection, sepsis, meningitis, hepatitis, and others. The diagnosis of complications was based on the treating physician’s documentation in the medical record. The treatment of influenza in the study hospital was based on the clinical practice guidelines of the Infectious Diseases Society of America^35^.

### Definition of endpoint

The primary endpoint was 30-day mortality^16,25,26,28,29,36^. Older persons who survived ≥30 days were considered “survivors” for this analysis. Telephone follow-up was used to ascertain 30-day survival if the patient was discharged before 30 days.

### Ethics statement

This study was conducted according to the Declaration of Helsinki. Because this was an observational study, the Cathay General Hospital Institutional Review Board approved the study protocol and waived the need for informed consent (written and oral) from the participants.

### Statistical methods

IBM SPSS Statistics version 23.0 for Mac (IBM, Armonk, NY, USA) was used to conduct all statistical analyses. The power was 0.82 using G-power 3.0 for analysis. Continuous data are presented as mean ± SD. An independent samples *t* test, or the Mann-Whitney-Wilcoxon test was used for continuous variables. Pearson’s chi-square test or Fisher’s exact test was used for categorical variables. Univariate variables with *p* < 0.1 were included in the multivariate stepwise (forward) logistic regression analysis for investigating independent mortality predictors^37^. The level of significance was set at 0.05 (two-tailed). Weights were assigned to each independent mortality predictor according to the predicted *b* values of the multivariate logistic regression analysis divided by 2 and rounded to the nearest integer^27^. Then, we developed a GID score and calculated the GID score for each patient. The stability of the GID score was evaluated by bootstrapping methods, for which we conducted random sampling from actual study patients, and 1000 hypothetical study populations were generated. We also estimated the bootstrapped effect size and 95% CI for each coefficient. The AUC was used to evaluate the discrimination of the score by bootstrap methods. We also used the Hosmer-Lemeshow test to evaluate goodness of fit.
